# Cancer caregivers unmet needs and emotional states across cancer treatment phases

**DOI:** 10.1371/journal.pone.0255901

**Published:** 2021-08-11

**Authors:** Winson Fu Zun Yang, Rachael Zhi Yi Lee, Sangita Kuparasundram, Terina Tan, Yiong Huak Chan, Konstadina Griva, Rathi Mahendran

**Affiliations:** 1 National University of Singapore, Singapore, Singapore; 2 Department of Psychological Science, College of Arts and Sciences, Texas Tech University, Lubbock, Texas, United States of America; 3 SingHealth Residency, Ministry of Health Holdings, Singapore, Singapore; 4 Department of Psychological Medicine, National University Hospital, Singapore, Singapore; 5 Lee Kong Chian School of Medicine, Imperial College & Nanyang Technological University, Singapore, Singapore; Hong Kong Polytechnic University, HONG KONG

## Abstract

**Study objective:**

To investigate the association between family cancer caregivers’ unmet daily needs and emotional states of depression, anxiety and stress across their care recipient’s treatment phases.

**Method:**

A cross-sectional study design and self-report questionnaires were used. Family caregivers (N = 237) of cancer patients in ambulatory cancer clinics were recruited from May to December 2017, and completed a sociodemographic and medical questionnaire, the Depression Anxiety Stress Scale and Needs Assessment of Family Caregivers-Cancer Scale. Hierarchical linear regression was conducted to examine the influence of each predictor (sociodemographic variables, unmet personal care and role management needs, cancer treatment phase) on the Depression Anxiety Stress Scale total score, depression subscale, anxiety subscale, and the stress subscale.

**Results:**

Family caregivers’ unmet daily activity needs, in particular higher unmet personal care needs, during the intermediate phase (6–9 months), were significantly associated (*ps*<0.05) with overall distress (*b* = 4.93) and stress (*b* = 2.26). In the chronic treatment phase (>9 months), the significant association of unmet personal care needs was with overall distress (*b* = 5.91), anxiety (*b* = 1.97) and stress (*b* = 2.53). After completing treatment, unmet role management needs were only significantly associated with stress (*b = -1*.*59*). Caregivers’ higher depression was also associated with greater unmet role management needs, regardless of treatment phases.

**Conclusions:**

Intermediate and chronic cancer treatment phases were identified as having greatest effect on caregivers’ unmet daily activity needs and emotions. Unmet personal care needs played the major effect on overall negative emotional states in the intermediate treatment phase and stress in the chronic treatment phase. Close attention to caregivers needs in intermediate and chronic treatment phases, would be highly beneficial in alleviating negative emotional disturbances.

## Introduction

Caring for cancer patients is associated with elevated stress levels which may negatively affect family caregivers’ physical health, psychological wellbeing, quality of life, and social functioning [1, 2]. Family cancer caregivers (FCCs) also experience depression (42.5%, range 33.31%– 51.29%) and anxiety (46.55%, range 35.59%– 57.52%) [3, 4]. These emotions are associated with different caregiver unmet needs in areas of healthcare service and information, psychosocial, financial, communication and personal/daily activity, which can impact patient care [4–8]. A caregiver’s need is defined as ‘unmet’ when an action or resource is needed to attain optimal well-being but is not satisfied [9]. Identifying these needs during the cancer patient’s treatment journey is a crucial first step in supporting caregiving efforts.

While the association of unmet caregiver needs and mood symptoms is known, there is limited evidence of when caregivers experience these symptoms along the cancer journey [10, 11]. Even the survivorship phase has shown a consistent association between psychosocial unmet needs and poorer caregiver mental health [12]. The existing research however, has methodological differences from cancer types, questionnaires used to assess caregiver needs and classifications of needs, making it difficult to estimate unmet needs, and thus requiring context-specific studies [13]. The few studies which have examined specific unmet needs such as perceived information needs, also found associations with higher odds of caregiver anxiety (OR = 3.86) and depressive (OR = 3.83) symptoms [8].

Of the different unmet needs FCCs have, daily activity unmet needs have a much wider impact with loss of quality sleep, daytime fatigue, interruptions to work life balance, and poorer social support [12, 14]. Daily activity unmet needs refer to needs that are associated with daily living such as personal care and care giving role management [12]. Daily activity needs are important to FCCs as a cancer patient consistently requires caregiver support, leading to very little personal time for FCCs [15]. For example, FCCs reduced their working hours or quit their jobs to care more for their loved ones, eventually affecting their work-life-caregiving balance [16]. Moreover, FCCs face internal conflict with regards to daily activity—if they do not take personal breaks from caregiving, their physical and mental health will deteriorate [17]; if they do take personal breaks from caregiving, they may experience guilt for doing so [18].

Little however, is known about patient treatment phases as a moderator of caregiver daily activity unmet needs and negative emotions. Kim and colleagues [19] reported that caregiving stress and lack of social support is associated with depressive symptoms within patients’ first year of diagnoses, but they did not examine the effects of specific treatment phases (i.e., early, later or completed treatment phases). Research has shown the high psychological impact on FCCs, at close to diagnosis, before patient’s first treatment and 6 months later [20, 21]. FCC depression was also noted to decrease at six months of treatment, suggesting caregiver adaptation to the situation or the stabilization of patient’s condition [22].

The aim of this study was to investigate the association between daily activity unmet needs of FCCs and negative emotional symptoms (stress, anxiety, and depressive symptoms) as a factor of cancer patients’ treatment phases (<6 months, 6–9 months, ≥9 months, or completed since start of treatment). We chose these time periods as they were critical to FCCs’ psychological health [19, 20, 23, 24]. The hypothesis was that FCCs’ daily activity unmet needs would be associated with higher negative emotional symptoms in the early stages (i.e. <6 months and 6-to-9 months) than in later stages (>9 months, and/or completed treatment) of cancer treatment.

## Materials and methods

FCCs (N = 237), defined as persons with familial connections (eg, spouse, parent, sibling, child, grandparent or others) and currently providing care to a patient with cancer, were recruited (May to December 2017) from ambulatory clinics at the National University Cancer Institute Singapore (NCIS). Participants were enrolled if they: 1) were Singapore citizens or permanent residents aged between 21 and 84 years, and 2) able to read and understand either English or Mandarin. The study had ethics approval (National Healthcare Group Domain Specific Review Board, Ref No. 2017/000/29) and Clinical Trial Registration (NCT03081312). Upon written informed consent, participants completed the self-report questionnaire at home.

The sociodemographic data collected included the FCC’s age, sex, marital status, and relationship with care recipient. Clinical/medical information of their care recipient, including type of cancer, cancer stage, and type of treatment and treatment duration were also gathered. For the purpose of this study, cancer patients’ treatment durations are grouped into four groups based on whether they have completed or still undergoing treatment since the start of treatment. Patients who answered “completed treatment” are grouped as “completed” treatment phase. In contrast, patients who have not completed treatment (i.e., currently receiving treatment at time of survey) are grouped into “<6 months”, “6–9 months”, and “>9 months”. Hence, we created a total of four categories of treatment phases: “<6months (early)”, “6–9 months (intermediate)”, “>9 months (Chronic)” and “completed”.

Caregiving needs, in the context of cancer care, was measured using the **Needs Assessment of Family Caregivers-Cancer (NAFC-C) Scale** [12]. This 27-item scale measures different caregiver needs on two dimensions: the importance of the need and the satisfaction with the fulfilment of the need during the past four weeks, and on a five-point Likert-type scale ranging from 0 (Not at all), to 4 (Extremely). The satisfaction rating was reverse coded. Each item was scored by multiplying the satisfaction rating (reversed coded) by the importance rating, yielding a range of 0 to 16, with higher score indicating a higher index of unfulfillment. The scale consists of four factors: 1) psychosocial unmet needs, 2) medical unmet needs, 3) financial unmet needs, and 4) daily activity unmet needs. These four factors are further divided into eight subscales. The NAFC-C has been previously validated and shown good reliability in an Asian population [25]. Of interest in this study are the subscales of the daily activity unmet needs, which examined caregiver personal care (‘getting help from others in order to take time for yourself’), and role management for survivor care (‘balancing work/school with caring for him/her’). Cronbach’s alpha for the ‘caregiver personal care’ subscale in this sample was acceptable (α = 0.74); for the ‘role management for survivor care’ subscale, Cronbach’s alpha was questionable (α = 0.68). Our previous validation study found that the NAFC-C adopted a different factor structure in an Asian population compared to the United States population. However, we intend to retain the subscales of daily activities to investigate the relationship between unmet daily activity needs and emotional states in FCCs.

Emotional states of depression, anxiety and stress were measured with the 21-item Depression Anxiety Stress Scale (DASS-21) [26]. Items are measured on a four-point Likert-type scale ranging from 0 (Never) to 3 (Almost always). Total, and subscale scores (for depression, anxiety, and stress) are computed by aggregating the scores of the items in the whole scale or respective subscales. Higher scores indicate higher depressive, anxiety, or stress symptoms. Good reliability and validity of this scale has been demonstrated in an Asian population [27]. In this sample, Cronbach’s alpha was excellent for the DASS total score (α = 0.94), and good for the depression (α = 0.88), anxiety (α = 0.84), and stress (α = 0.86) subscales.

### Statistical analysis

Participant group demographics, and psychosocial variable scores (DASS-21, unmet personal care, and unmet role management needs) were analysed using chi-square test of independence, and several one-way analysis of variances (ANOVAs). Covariates that were included in the subsequent analyses were age group and cancer stage. Unmet personal care and role management needs and their subscales were centred to compute the interaction term between these subscales and care recipient treatment phases. Hierarchical linear regression was conducted to examine the influence of each predictor at each step of the analysis on the DASS a) total score; b) depression subscale, c) anxiety subscale, and d) stress subscale. The first step consisted of only the covariates. The variables of interest, i.e. personal care, role management, care recipient’s treatment phase, and their interactions, were added into the second step. The two steps were compared using a general linear model approach. All analyses were conducted in R 3.4.3 loading on R Studio 1.1.423.

## Results

Socio-demographic characteristics of the FCCs and medical characteristics of their care recipients are presented in Table 1.

**Table 1:** Participants demographics from different treatment phases.

N (%)	<6 months	6–9 months	>9 months	Completed
	(n = 77)	(n = 42)	(n = 34)	(n = 84)
**Sex**				
Female	47 (61.00%)	21 (50.00%)	20 (58.80%)	55 (65.50%)
Male	30 (39.00%)	21 (50.00%)	14 (41.20%)	29 (34.50%)
**Age group**				
21–30	17 (22.10%)	3 (7.10%)	2 (5.90%)	15 (17.90%)
31–40	21 (27.30%)	12 (28.60%)	4 (11.80%)	13 (15.50%)
41–50	19 (24.70%)	13 (31.00%)	8 (23.50%)	19 (22.60%)
51–60	14 (18.20%)	8 (19.00%)	7 (20.60%)	22 (26.20%)
61–70	4 (5.20%)	6 (14.30%)	11 (32.40%)	13 (15.50%)
71–80	2 (2.60%)	0 (0.00%)	2 (5.90%)	2 (2.40%)
**Marital status**				
Not married	31 (40.30%)	10 (23.80%)	7 (20.60%)	35 (41.70%)
Married	46 (59.70%)	32 (76.20%)	27 (79.40%)	49 (58.30%)
**Relationship with care recipient**				
Your spouse	21 (27.30%)	14 (33.30%)	17 (50.00%)	26 (31.00%)
Your child	4 (5.20%)	0 (0.00%)	1 (2.90%)	5 (6.00%)
Your grandparent	3 (3.90%)	0 (0.00%)	1 (2.90%)	3 (3.60%)
Your parent	41 (53.20%)	23 (54.80%)	12 (35.30%)	42 (50.00%)
Sibling	8 (10.40%)	5 (11.90%)	3 (8.80%)	8 (9.50%)
**Cancer type**				
Brain tumor	2 (2.60%)	0 (0.00%)	0 (0.00%)	2 (2.40%)
Breast	12 (15.60%)	13 (31.00%)	9 (26.50%)	20 (23.80%)
Gastro-intestinal/Colorectal/Stomach	16 (20.80%)	5 (11.90%)	2 (5.90%)	20 (23.80%)
Gynaecological	2 (2.60%)	2 (4.80%)	3 (8.80%)	2 (2.40%)
Haemotological/Leukemia/Lymphoma/Myeloma	6 (7.80%)	4 (9.50%)	7 (20.60%)	7 (8.30%)
Lung	15 (19.50%)	8 (19.00%)	9 (26.50%)	18 (21.40%)
Multisite	1 (1.30%)	3 (7.10%)	0 (0.00%)	3 (3.60%)
NPC/Throat/Oral	1 (1.30%)	2 (4.80%)	1 (2.90%)	5 (6.00%)
Others	15 (19.50%)	2 (4.80%)	2 (5.90%)	3 (3.60%)
Pancreas	6 (7.80%)	0 (0.00%)	0 (0.00%)	3 (3.60%)
Renal	1 (1.30%)	3 (7.10%)	1 (2.90%)	1 (1.20%)
**Cancer stage of care recipient**				
Early (stages 0–2)	10 (13.00%)	5 (11.90%)	6 (17.60%)	28 (32.30%)
Late (stages 3–4)	67 (87.00%)	37 (88.10%)	28 (82.40%)	56 (66.70%)
**Depression, Anxiety, and Stress (SD)**				
Total	10.95 (10.57)	7.40 (9.06)	10.09 (8.49)	10.80 (8.93)
Depression	3.52 (4.08)	2.10 (3.20)	2.88 (2.78)	3.01 (3.22)
Anxiety	2.83 (3.48)	1.57 (3.05)	2.85 (3.12)	3.06 (3.26)
Stress	4.60 (3.87)	3.74 (3.58)	4.35 (3.31)	4.73 (3.66)
**Daily activities (SD)**				
Personal roles	1.88 (1.89)	1.16 (1.71)	1.62 (2.02)	1.71 (2.08)
Role management	2.11 (2.51)	1.95 (1.74)	1.87 (2.52)	1.79 (2.62)

Between care recipients’ treatment phase, chi-square test of independence revealed a difference in distribution of age group (*χ*^*2*^*(*15) = 28.46, *p* = 0.019) and cancer stages (*χ*^*2*^(3) = 14.12, *p* = 0.0028). Later cancer stages were most prevalent in care recipients who had <6 months of treatment (*n =* 67, 87.01%), 6–9 months of treatment (*n =* 37, 88.10%), >9 months of treatment (*n =* 28, 82.35%), and those who have completed treatment (*n =* 56, 66.88%). There was no significant distribution of sex (χ^2^(3) = 3.02, p>0.05), relationship with care recipient (χ^2^(12) = 10.05, p>0.05), and marital status (χ^2^(3) = 7.79, p>0.05).

One-way ANOVAs revealed no group differences between care recipient’s treatment phases on personal needs (*p*>0.05), role management (*p*>0.05), DASS (*p*>0.05) or any of their subscales (*ps*>0.05).

Main Regression: In the first step, participant’s characteristics were not associated with any of the depressive scores (*ps*>0.05) except for age 51–60 compared to age 21–30 on anxiety (b = 1.45, *p* = 0.042). Adding unmet personal care and role management needs, treatment phases and their interaction in step 2 resulted in a significant model change for DASS total score *(R*^*2*^ change = 0.19, *F*(11, 220) = 4.65, *p*<0.001), depression score (*R*^*2*^ change = 0.19, *F*(11, 220) = 4.71, *p<*0.001), anxiety score (*R*^*2*^ change = 0.13, *F*(11, 220) = 3.21, *p*<0.001), and stress score (*R*^*2*^ change = 0.18, *F*(11, 222) = 4.30, *p*<0.001). Details of the model and coefficients are displayed in Table 2.

**Table 2:** Unstandardized regression coefficients of predictors of depressive symptoms.

Unstandardized b (SE)	DASS	DASS	DASS	DASS
	Total	Depression	Anxiety	Stress
**Step 1: covariates**	R^2^ = 0.033	R^2^ = 0.031	R^2^ = 0.047	R^2^ = 0.024
Intercept	10.92 (1.88)***	3.55 (0.69)***	2.77 (0.65)***	4.60 (0.73)***
Age group (21–30 as reference)				
31–40	-1.86 (2.07)	-0.97 (0.76)	0.06 (0.71)	-0.95 (0.8)
41–50	-3.07 (1.98)	-1.42 (0.73)	-0.54 (0.68)	-1.11 (0.77)
51–60	1.49 (2.04)	-0.11 (0.75)	1.45 (0.70)*	0.16 (0.79)
61–70	-2.20 (2.22)	-1.40 (0.82)	0.04 (0.77)	-0.85 (0.86)
71–80	-1.45 (4.15)	-1.27 (1.52)	0.64 (1.43)	-0.81 (1.61)
Cancer Stage (early as reference)				
Late (stages 3–4)	0.54 (1.52)	0.33 (0.56)	-0.37 (0.52)	0.57 (0.59)
**Step 2: Main + interaction effects**	R^2^ = 0.22***	R^2^ = 0.22***	R^2^ = 0.18***	R^2^ = 0.20***
Personal care	0.75 (1.70)	0.26 (0.62)	0.53 (0.60)	-0.04 (0.66)
Role management	2.80 (1.58)	1.32 (0.58)*	0.42 (0.56)	1.06 (0.62)
treatment phase (<6 months as reference)				
6–9 months (intermediate)	-1.75 (1.75)	-0.73 (0.64)	-0.83 (0.62)	-0.18 (0.68)
>9 months (chronic)	-1.08 (1.88)	-0.54 (0.69)	-0.19 (0.67)	-0.35 (0.73)
completed	-0.48 (1.44)	-0.55 (0.53)	0.00 (0.51)	0.06 (0.56)
Personal care x treatment phase (DA x<6 months as reference)				
Personal care x 6–9 months	4.93 (2.37)*	1.66 (0.87)	1.01 (0.84)	2.26 (0.93)*
Personal care x >9 months	5.91 (2.75)*	1.42 (1.01)	1.97 (0.98)*	2.53 (1.07)*
Personal care x completed	2.06 (2.04)	1.00 (0.75)	-0.16 (0.73)	1.22 (0.80)
Role management x treatment phase (DA x<6 months as reference)				
Role management x 6–9 months	-2.74 (2.55)	-1.29 (0.93)	-0.18 (0.91)	-1.26 (1.00)
Role management x >9 months	-4.49 (2.68)	-1.51 (0.98)	-1.07 (0.96)	-1.91 (1.05)
Role management x completed	-2.89 (1.96)	-1.59 (0.72)*	-0.55 (0.70)	-0.75 (0.76)

DASS: Depression Anxiety Stress Scale; DA: Daily activity

* p<0.05,

** p<0.01,

*** p<0.001

**Fig 1:**
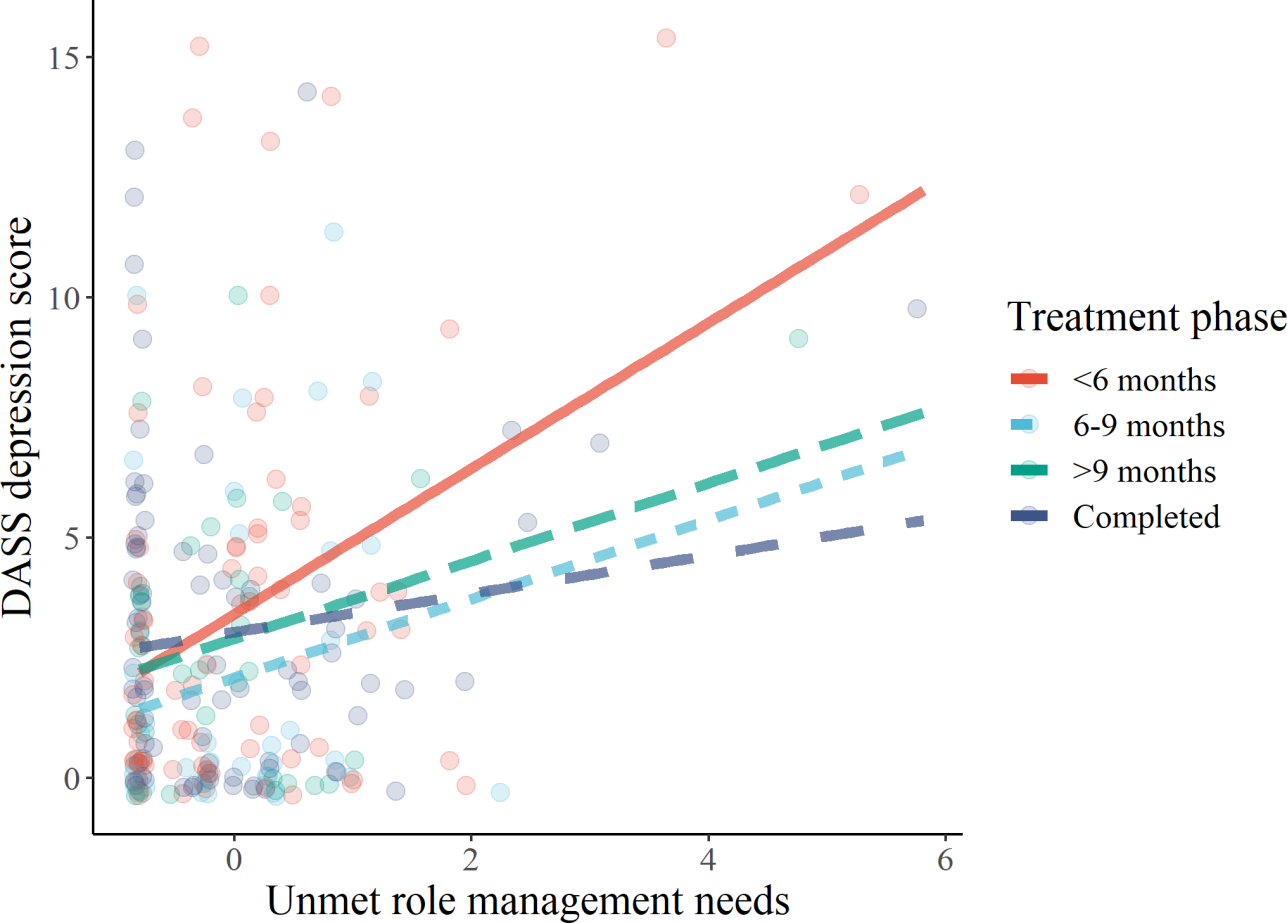
Relationship between unmet role management needs and DASS depression score across treatment phases. Greater unmet role management needs were associated with greater Depression, Anxiety, and Stress Scale (DASS) depression score in general. Moreover, compared with their loved ones undergoing treatment for less than six months, family caregivers with care recipient who have completed treatment had less positive relationship between unmet role management needs and DASS depression score.

In short, greater unmet personal care needs among participants whose care recipients have been undergoing treatment for 6 to 9 months compared to less than 6 months was related to higher DASS total score and stress score. Additionally, greater unmet personal care needs among participants whose care recipients have been undergoing treatment for more than 9 months compared to less than 6 months was related to higher DASS total, anxiety, and stress scores. Greater unmet role management needs among participants whose care recipients have been undergoing treatment for more than 9 months compared to less than 6 months was related to lower depression score. Additionally, greater unmet role management needs was related to greater depression score (Fig 1).

## Discussion

This study investigated the moderation effects of care recipients’ treatment phases on their FCCs daily activity unmet needs and three related negative emotional states of depression, anxiety and stress. Firstly, compared to care recipient’s early treatment phase (<6 months), in the care recipient’s intermediate (6–9 months) phase, a significant association was found between FCC’s unmet personal care needs and higher overall distress and stress. In the longer term, (>9 months, chronic treatment phase), higher unmet personal care needs were associated with significantly higher overall distress, anxiety and stress, but not depression. Secondly, in general, higher unmet role management needs was associated with only higher overall distress. Moreover, compared with care recipient’s early treatment phase (<6 months), in care recipient’s completed treatment phase, the relationship between unmet role management needs and depression was positive, although the slope was gentler.

### Daily activity unmet need and negative emotional states

Overall, we found that daily activity needs were significantly associated with overall distress, stress, depressive and anxiety symptoms. This is similar to previous findings that FCCs’ unmet day-to-day needs such as higher interference in regular activities or looking after one’s own health were associated with depression and anxiety [23, 28]. Kim et al. [12] found that while the prevalence of caregivers’ daily activity unmet needs decreases over time in the survivorship phase from 50% at 2-month to 23.7% after 5-years of survivorship, personal daily activity needs remained unmet. Others found that unmet information needs and psychosocial/emotional needs predicted higher psychological distress [23, 29]. However, as research to date has involved different instruments it is still not possible to identify the most prevalent unmet need(s) associated with negative emotional states. Our findings however highlight the importance of FCC daily activity unmet needs in contributing to negative emotional states during cancer treatment phases.

We further demonstrated differential patterns of unmet daily activity needs: 1) FCCs with care recipients in the intermediate treatment phase had stress-related distress and this was associated with personal care, 2) FCCs with care recipients in the chronic treatment phase had an additional anxiety-related distress, and 3) the relationship between unmet role management needs and depression are similar across FCCs with care recipients who are still undergoing treatment.

### Intermediate treatment phase

Other studies have found higher depression and anxiety in the initial treatment phase and terminal stages of cancer. In early or acute treatment phases, medical and financial issues often over-ride the psychological issues FCCs face [2]. They are focused on medical and financial challenges in the acute treatment phase and supporting and attending to their lived one, and may ignore their own personal care needs [30]. As cancer treatment progresses, changes arise in FCCs daily lives and they become more aware of their own health [10, 19]. Our study supports this change and views the intermediate treatment phase as a transitional period when the FCCs, who might have earlier subsumed their own needs, now begin to adjust to the caregiving role and need to attend to their own needs [19, 31].

Additionally the finding of the significant association of unmet personal care needs with depression and stress during the intermediate treatment phase addresses the gap in the literature where only early treatment phases such as at the point of diagnosis or treatment were examined [4, 31]. As FCCs transition to a caregiving role, much of their personal and social time is sacrificed for caregiving, and emotional adaptations are required; this mimics findings in caregivers of dementia and stroke patients [32, 33]. The presence of respite care and social support is vital in enabling FCCs to cope with the new responsibilities and changes in their lives.

### Chronic treatment phase

The finding that FCCs with unmet daily activity needs was significantly associated with stress and anxiety but not depression in the chronic treatment phase is consistent with earlier research findings of increasing anxiety over time and with depression, less long lasting [34]. Hence screening for anxiety in FCCs during this period should not be overlooked.

While this study did not find any significant contribution of unmet daily activity needs pertaining to role management on psychological distress during the chronic treatment phase, the contribution of this association, however, approached significance levels. The results still hold valuable insights into FCCs’ needs and psychological well-being. According to the role strain theory, caregiver and work responsibilities frequently compete, and conflict happens when individuals struggle to meet demands from these competing roles [35]. Previous studies have also shown that FCCs quit or lose their jobs due to changing responsibilities, which results in depression [36]. A similar trend towards depression with FCCs changing roles and responsibilities was noted in this study. FCCs could be anxious as they transition into long-term care as they may be unsure of future or long-term responsibilities and changes [37].

Yet the association was not significant possibly as FCCs may have already transitioned well into the primary caregiving role; one might speculate that it also takes time to settle into new roles [38]. The data presented in this study suggests that this may be the case. Primary concerns of FCCs may be personal needs, which was evident during the intermediate treatment phase. As role management requires communication within the family, these issues may have been resolved naturally when the FCC’s personal needs were discussed within the family and met later. At this juncture, no definite conclusions can be made about the influence of role management on depressive symptoms during the chronic treatment phase. More power would be needed to confirm its effect on psychological distress.

### Role management during and after treatment period

Our data revealed that caregivers continually face stress during treatment periods and this was associated with increased unmet role management needs. After treatment has been completed, FCCs still face higher depression with greater unmet role management needs, although this relationship was lesser than when treatment first started. This was similar to a previous study where husbands were assisting their wives less after their wives completed treatment for breast cancer [39]. After treatment has been completed, FCCs may have more time to adjust their roles such as work-life balance, family roles, and assisting their loved ones with daily chores or routines. In other words, they are able to relax a little and allow some more time to manage other personal responsibilities [40]. However, even as they settle into a “new normal” after sacrificing their jobs, time, and space [41], having to find a new stable job if they had previously quit their job or facing added responsibilities in their current one, exacerbates residual problems leading to depression. Therefore, this time period is a secondary transition period for FCCs [41].

### Implications

Our data raises several implications for clinicians and healthcare administration. First, given that the intermediate treatment phase is critical for a healthy mental state, there is an immediate need to promote or encourage FCCs to allocate some personal time for themselves before the burden and distress accumulates and transfers into the chronic stage. FCC’s well-being directly affects that of the patient [11, 36, 42] and if FCCs’ needs are met and they would be in a better position to care and support their loved ones [15]. Interventions and support systems need to be set in place for FCCs, especially during the early treatment phase. Education on achieving a work-life-caregiving balance, could help FCCs learn to balance their time, manage their roles, and attend to their self-care [43]. Next, clinicians could also informally assess how the caregiver is coping during patient consultations, so that their needs are recognised and addressed [44]. Studies however have shown that although interventions and social support already exist, and FCC express interest in utilizing these many actually do not avail themselves of the services [45]. This may be due to a lack of motivation or lack of resources to guide FCCs to the right avenues. Therefore, clinicians could be that bridge in introducing such strategies in the early caregiving stage for those who report some form of distress.

There are several limitations, foremost of which is the cross-sectional study design which precludes conclusions about changes in associations of the negative emotions and daily activity unmet needs in FCCs across cancer treatment phases. The sample size is small, particularly when the different treatment phases were analysed. A second-level clinician interview for caregivers’ with high negative emotions would have provided a definitive assessment of the self-reported emotional state. This study also did not examine the impact of the dynamic patient-caregiver relationship; care recipient’s attitudes, needs, reliance on the FCC would also have a significant effect on the latter’s needs and emotions. Future research should investigate FCCs’ transitioning process in the role of caregiving as this would help in supportive interventions and caregiving skills training. Finally, we did not investigate the effect of multiple cancer types on FCC’s on the relationship as our data is underpowered to do so. Finally, we acknowledge an important limitation as the study design included caregiving for different types of cancers which may be associated with different stressors and burden for caregivers. All cancer types were included as the number of subjects for each of the different cancer types was small.

Despite limitations, this study suggests several useful opportunities to prevent or alleviate anxiety, depression and stress in FCCs. Caregivers could be better supported by efforts firstly in screening of emotional states and unmet needs at regular intervals during the patient’s cancer journey and as care transitions from ambulatory or hospital care, to home settings [38]. Next, although causal links have yet to be established between activity needs and negative emotional states, the present findings suggest: 1) the need for interventions that could alleviate day-to-day needs such as respite care or social support, and 2) these interventions should be implemented early preferably in the first 6 months of care provision for better adaptation to long term care needs of the patient. Lastly, it is important to emphasize the need for FCCs to prioritize self-care and learn to identify and attend to their own needs during the caregiving journey.

## Conclusion

This study highlights the vulnerability and risk of unmet needs and negative emotional states in FCCs during critical periods of their care recipient’s cancer journey. The intermediate treatment phase of 6–9 months was identified as a period of high risk of unmet needs and distress, anxiety and depression, with personal care being important. In the chronic treatment phase, anxiety rather than depression continues to be a risk for FCCs with unmet needs. Although causal links and directions are still unclear, the study highlights the need for greater attention to FCCs needs.
